# Successful Pregnancy after Treatment with Ulipristal Acetate for Uterine Fibroids

**DOI:** 10.1155/2014/314587

**Published:** 2014-07-21

**Authors:** Javier Monleón, Alicia Martínez-Varea, Daniela Galliano, Antonio Pellicer

**Affiliations:** ^1^Department of Obstetrics and Gynecology, La Fe University Hospital, Bulevar Sur s/n, 46026 Valencia, Spain; ^2^Instituto Valenciano de Infertilidad (IVI), 08017 Barcelona, Spain

## Abstract

This case report presents a clinical pregnancy after ulipristal acetate (UA) to decrease uterine fibroid size. A 37-year-old patient, gravida 1, abortus 1, with uterine fibroids was treated with 5 mg of UA daily for 13 weeks starting eight months after a multiple laparotomic myomectomy. Fibroid shrinkage and restoration of the morphology of endometrial cavity were evaluated in order to allow a subsequent pregnancy. A decrease of the uterine fibroids and a normal morphology of the endometrial cavity were noted by transvaginal ultrasound after treatment. An endometrial biopsy excluded histologic endometrial changes. Three months after the end of UA the patient reported amenorrhea for 5 weeks and a clinical pregnancy was confirmed with transvaginal ultrasound. She underwent a subsequent uneventful pregnancy. Thus, the spontaneous pregnancy after UA to reduce fibroid size may support the potential clinical utility of this selective progesterone receptor modulator in the management of women with pregnancy desire and uterine fibroids after a prior myomectomy. Patients who refuse a new surgical procedure and/or those who are going to undergo assisted reproductive techniques would benefit from UA. It effectively shrinks fibroids, avoids risks of a new surgical procedure, and allows an immediate attempt at conception after the end of treatment.

## 1. Introduction

Uterine fibroids, or leiomyomas, are benign uterine neoplasms that arise from the smooth-muscle tissue [1]. They constitute the most common tumor in women [1, 2], being present in 20 to 40% of women of reproductive age [2, 3]. Related symptoms depend on their size and uterine location [1, 4]. It appears that submucosal and intramural fibroids are associated with decreased fertility and an increased miscarriage rate [4, 5]. The management of uterine fibroids in women with reproductive desire is still controversial [4, 6]. Whereas myomectomy for submucosal and intramural fibroids may improve fertility outcomes, current evidence is insufficient to determine the effect of radiologic interventions in fertility [4]. Finally, the role of the few available alternative medical therapies [4, 7] in patients desiring future fertility is limited. Thus, medical treatments are not advocated for managing these women [4].

Ulipristal acetate is a selective progesterone receptor modulator (SPRM) already approved for the treatment of uterine fibroids in Europe [8]. The effectiveness of ulipristal acetate in doses of either 5 mg or 10 mg daily for 13 weeks has been demonstrated to control excessive bleeding and to reduce the size of fibroids [3]. It is also as effective as leuprolide acetate for controlling uterine bleeding, but with significantly fewer hot flashes [7]. Data on the benign histologic endometrial changes induced by ulipristal acetate are reassuring, as they tend to be spontaneously resolved within a few months after the end of the 13-week treatment [9, 10]. Effectiveness and safety of medium-term use of ulipristal acetate has recently been shown. After four repeated intermittent 3-month courses of ulipristal acetate 10 mg daily, high rates of amenorrhea and further fibroid shrinkage have been described, and no cases of endometrial hyperplasia or adenocarcinoma have been reported [9].

There is a lack of evidence regarding the usefulness of ulipristal acetate to decrease fibroid size in order to avoid its impact on the endometrial cavity prior to pregnancy [5, 11]. We report a case of a successful spontaneous pregnancy after ulipristal acetate to reduce the size of uterine fibroids. This approach may constitute a new paradigm in the management of fibroids in infertility.

## 2. Materials and Methods

### 2.1. Patient Information

A 37-year-old woman with an unremarkable past medical and surgical history came to our gynecological clinic with lower abdominal pain. She had one pregnancy loss one year before. Transvaginal ultrasound revealed seven intramural uterine fibroids, with maximum diameters between 22 and 82 mm. Since the endometrial cavity was distorted by the fibroids, the patient underwent a laparotomic myomectomy, in which seven myomas were removed and one endometrial disruption was sutured. Both adnexa appeared normal. Her postoperative course was uneventful.

### 2.2. Ulipristal Acetate Administration for Fibroid Shrinkage

Eight months following the surgical procedure, the patient reported to have normal menstrual periods and she desired to become pregnant. Nevertheless, transvaginal ultrasound showed two intramural fibroids with maximum diameters of 21 and 25 mm, respectively. They were located in the posterior uterine wall and slightly deformed the endometrial cavity. The patient was therefore treated with a 5 mg daily dose of ulipristal acetate for 13 weeks. Pearls and pitfalls of the treatment were explained to the woman, and her informed consent was obtained.

### 2.3. Follow-Up Examinations

Monthly follow-up visits were carried out. The patient persisted with amenorrhea from the beginning of the treatment, and no side effects were reported following treatment.

One month after the end of the treatment, she reported to have had one normal menstruation. Transvaginal ultrasound revealed a decrease in the two uterine fibroids to maximum diameters of 15 and 21 mm, respectively, and a normal morphology of the endometrial cavity. Actually, an endometrial biopsy obtained in the 22nd day of the menstrual cycle, before her second menstruation, revealed a normal secretory endometrium.

## 3. Results

Three months after ending ulipristal acetate, the patient returned to the clinic complaining of amenorrhea for 5 weeks. Using transvaginal ultrasound, both intrauterine pregnancy and unmodified uterine fibroids were seen. The pregnancy was uneventful and subsequent obstetric ultrasounds revealed a discreet increase of the two uterine fibroids, with diameters of 27 and 45 mm, respectively, on the 28th week of pregnancy. The endometrial cavity did not appear to be distorted by the fibroids. An elective cesarean section was performed at 34 weeks of pregnancy because the patient had regular uterine contractions and prior myometrial damage with endometrial disruption during a laparotomic myomectomy in which seven myomas were removed. The healthy female newborn weighed 2432 g. Both the mother and the child were discharged 48 hours following the surgery, after an uncomplicated immediate postpartum period.

## 4. Discussion

Uterine fibroids are present in approximately 70% and 80% of 50-year-old white and black women, respectively [1, 2]. Common symptoms include heavy menstrual bleeding and subsequent anemia, pelvic pain, dysmenorrhea, decreased quality of life, and reproductive dysfunction [4, 7, 9].

The optimal treatment for patients with symptomatic uterine fibroids and pregnancy desire remains unknown [3, 4, 6, 8]. It has been reported that myomectomy may improve fertility outcomes in women with submucosal and intramural fibroids [4]. Nevertheless, there is still insufficient evidence from randomised controlled trials to establish the effect of myomectomy to improve fertility [6]. On the other hand, current evidence is still insufficient to establish whether radiologic procedures represent a valid treatment option for women with symptomatic fibroids who want to preserve their fertility [4]. Alternative medical therapies have limitations [7] and are not considered a valid fertility-preserving treatment option [4]. Uterine fibroid growth depends on the ovarian steroids estrogen and progesterone [1]. Accordingly, oral progestin may promote fibroid growth and induce abnormal bleeding [7]. Although the progestin-releasing intrauterine device would control heavy menstrual bleeding, it is hardly ever used in women with a deformed endometrial cavity by submucosal fibroids [7] and also prevents pregnancy if used. The gonadotropin-releasing hormone analogues (GnRHa) are the most effective medical treatment for fibroids-related symptoms [7]. Nevertheless, hot flashes are reported by 67% of users [7] and efforts to conceive may be delayed [4]. Other medical options that reduce fibroid volume include danazol, mifepristone, aromatase inhibitors, and raloxifene. However, their benefit in patients with pregnancy desire is still unclear [4].

Treatment with 5 mg or 10 mg of the SPRM ulipristal acetate for 13 weeks controls excessive bleeding and reduces total fibroid volume [3]. These regimens are as effective as leuprolide acetate in controlling uterine bleeding and induce significantly fewer hot flashes [7]. Although leuprolide acetate induces significantly greater uterine volume shrinkage than ulipristal acetate, the SPRM provides a more sustained effect on the reduction of myoma volume after discontinuation of treatment [7]. It has recently been shown that four repeated intermittent 3-month courses of ulipristal acetate 10 mg daily induce both a higher rate of amenorrhea and a greater median fibroid volume reduction, compared with a single ulipristal acetate course. Interestingly, a sustained fibroid volume shrinkage at 3 months after the last ulipristal acetate course has also been reported [9].

Either a 5 mg or 10 mg daily dose of ulipristal acetate for 13 weeks has been associated with benign endometrial changes that are resolved within 6 months of treatment [3]. Whereas simple endometrial hyperplasia has been shown in 28% of those using these regimens for 6 months [12], it was not found in another study with ulipristal acetate 5 mg daily for 26 weeks [13]. Interestingly, neither endometrial hyperplasia nor adenocarcinoma has been reported in four repeated intermittent 3-month courses of ulipristal acetate 10 mg daily [9]. Moreover, no premalignant findings have been found in cynomolgus monkeys chronically exposed to up to 150 times the clinical UA exposure [14]. Therefore, available data is encouraging regarding the safety of the long-term use of ulipristal acetate for uterine fibroids.

It is known that the sharp increases and decreases of estrogen and progesterone during pregnancy and the postpartum period have severe consequences for fibroid growth [1]. On the other hand, abdominal myomectomies have an associated risk of subsequent pelvic adhesions that may have a detrimental impact upon fertility [4]. There is also still insufficient evidence to establish that myomectomy improves fertility [6]. Moreover, the endometrial damage during myomectomy may lead to a caesarean section in future deliveries [4]. It is advisable for patients to delay conception at least three months after myomectomy [4]. Our patient had a remaining distorted endometrial cavity by intramural fibroids after an abdominal myomectomy, which might increase the possibility of a new miscarriage and decrease her fertility. It was also taken into account that there is a potential risk of fibroid growth during pregnancy. Thus, a conservative approach was carried out with ulipristal acetate in order to decrease fibroid size and restore the endometrial cavity, therefore avoiding the associated risks of a second uterine surgery and the subsequent delay of conception.

Ulipristal acetate 5 mg for 13 weeks successfully reduced fibroid size and allowed the reestablishment of the morphology of endometrial cavity. As soon as the anatomopathological study excluded endometrial changes after treatment, the patient was able to become pregnant. Three months after the end of ulipristal acetate treatment the woman came to our clinic at five weeks of spontaneous gestation and subsequently underwent an uncomplicated pregnancy. Since the patient had a previous endometrial disruption during a multiple laparotomic myomectomy, an elective cesarean section was carried out when she started labor.

The spontaneous pregnancy after ulipristal acetate to reduce fibroid size may support the potential clinical utility of this SPRM in the management of women with pregnancy desire and uterine fibroids after a prior myomectomy. In particular, patients who refuse a new surgical procedure and/or those who are going to undergo assisted reproductive techniques would benefit from this therapy. Since ulipristal acetate effectively shrinks fibroids and avoids risks of a new surgical procedure, it would allow an immediate attempt at conception at the end of treatment. Although no pregnancy-related complications or teratogenic effects have been reported to date, further series are required in order to establish the safety of ulipristal acetate as a treatment of symptomatic fibroids prior to pregnancy.
